# Highlight report: Relevance of T-cells, B-cells and immune checkpoint factors for prognosis of breast cancer

**DOI:** 10.17179/excli2018-2019

**Published:** 2019-04-11

**Authors:** Maiju Myllys

**Affiliations:** 1 IfADo - Leibniz Research Centre for Working Environment and Human Factors, Dortmund, GERMANY

## ⁯

Recently, Anne-Sophie Heimes and colleagues from the University Hospital of Mainz published a study to gain a better understanding of the association of specific immune responses with prognosis in breast cancer (Heimes et al., 2017[8]). Although it is well established that tumor-infiltrating lymphocytes have prognostic and predictive impact, the specific role of individual cell types is still discussed controversially (Schumacher and Schreiber, 2015[19]; Denkert et al., 2010[4]; Salgado et al., 2015[15]; Iglesia et al., 2016[10]; Rody et al., 2009[14]; Mahmoud et al., 2011[11]). To gain a deeper understanding, 197 node-negative breast carcinomas of patients not treated with adjuvant therapy were analyzed for T-cell and B-cell markers based on gene-expression data by immunostaining (Heimes et al., 2017[8]). Moreover, two immune checkpoint markers, PD-1 and CTLA-4, were analyzed. In a multivariate analysis, infiltration of both T-cells and B-cells was significantly associated with better prognosis. Also the immune checkpoint markers showed a significant association with prognosis, independent of the clinical-pathological variables. Particularly interesting results were obtained after analysis of the molecular subtypes HER2+, basal-like (ER-/ HER2-), luminal A (ER+, HER2-, AURKA-low) and luminal B (ER+, HER2-, AURKA-high). The prognostic effect of immune cells (T- and B-cells) was strongest in the HER2+ molecular subtype. Major differences were obtained between the other molecular subtypes with T-cells most pronounced in the luminal A, B-cells in the luminal B and the immune regulators in basal-like carcinomas.

The positive prognostic influence of lymphocytic infiltrates has been known for decades (Di Paola et al., 1974[5]; Aaltomaa et al., 1992[1]). While the relevance of T-cells has been accepted since long, the key prognostic impact of the humoral immune system has only been reported in 2008 (Schmidt et al., 2008[16]) and the prognostic role of individual cell types and related factor of influence have been further assessed in several studies (Heimes et al., 2017[8]; Schmidt et al., 2018[18], 2012[17]; Mattsson et al., 2015[13]; Sicking et al., 2014[20]). The situation remains challenging, since only the presence of T- and B-cells in tumor tissue does not seem to be sufficient to guarantee a favorable prognostic influence. Besides lymphocyte infiltration, further factors seem to be relevant, including the redox status, migration capacity, proliferation and the metabolic microenvironment (Hammad et al., 2016[6]; Cadenas et al., 2010[2], 2014[3]; Marchan et al., 2017[12]; Hassan et al., 2017[7]; Hellwig et al., 2016[9]; Stock et al., 2015[22]; Stewart et al., 2012[21]). A particularly critical aspect is to consider negative immune regulators, such as CTLA-4 and PD-1. In conclusion, the study of Heimes and colleagues (2017[8]) clearly shows that the analysis of tumor infiltrating lymphocytes should at least include differentiation between T-cells, B-cells/plasma cells and negative immune regulators, and should independently consider the four molecular subtypes HER2+, basal-like, luminal A and luminal B.
